# Supplementary material for: Patterns of Intron Gain and Loss in Fungi
Source: PLoS Biol. 2004 Nov 30;2(12):e422. doi: 10.1371/journal.pbio.0020422 (PMC532390; doi:10.1371/journal.pbio.0020422)
Supplement: Table S1 — Also available at http://genes.mit.edu/NielsenEtAl/. (4.3 MB ZIP). [file pbio.0020422.st001.zip › NielsenEtAl/html/792.html]

AN4501.1.NCU02806.1.MG01588.1.FG06847.1


```
 CLUSTAL W (1.82) Multiple Sequence Alignments - Introns Inserted


Sequence 1: NCU02806.1	262 aa
Sequence 2: MG01588.1	272 aa
Sequence 3: FG06847.1	272 aa
Sequence 4: AN4501.1	261 aa
Alignment Length: 273 aa
Number Identitical Residues: 214 aa
Alignment Score (without introns) 8105


MG01588.1 	MGHEDAVYLAKLAEQAERYEE1MVENMKIVASEDRDLTVEERNLLSVAYKNVIGARRASW
NCU02806.1	MGHEDAVYLAKLAEQAERYEE1MVENMKIVASEDRDLTVEERNLLSVAYKNVIGARRASW
FG06847.1 	MGHEDAVYLAKLAEQAERYEE1MVENMKIVAGEDRDLTVEERNLLSVAYKNVIGARRASW
AN4501.1  	MGHEDAVYLAKLAEQAERYEE1MVENMKVVAASDVELTVEERNLLSVAYKNVIGARRASW
          	********************* ******:**..* :************************

MG01588.1 	RIVTSIEQKEESKGNSSQVGLIKEYRTKIEAELAKICEDILSVLDKHLIPSAKSGESKVF
NCU02806.1	RIVTSIEQKEESKGNSTQVSLIKEYRQKIESELAKICEDILSVLDQHLIPSATTGESKVF
FG06847.1 	RIVTSIEQKEESKGNSSQVTLIKEYRQKIEAELAKICEDILEVLDQHLIPSAKSGESKVF
AN4501.1  	RIVTSIEQKEESKGNESQVALIKEYRQKIEGELAKICQDILDVLDQHLIKSAQSGESKVF
          	***************.:** ****** ***.******:***.***:*** ** :******

MG01588.1 	YHKM2KGDYHRYLAEFAIGDKRKDSADKSLEAYKAATEVAQTELPPTHPIRLGLALNFSV
NCU02806.1	YHKM2KGDYHRYLAEFAVGDKRKDSADKSLDAYKNATDVAQTELPPTHPIRLGLALNFSV
FG06847.1 	YHKM2KGDYHRYLAEFAIGDRRKDSADKSLEAYKAATEVAQTELPPTHPIRLGLALNFSV
AN4501.1  	YHKM2KGDYHRYLAEFAVGDNRKAAADASLEAYKSATEVAQTDLAPTHPIRLGLALNFSV
          	**** ************:**.** :** **:*** **:****:*.***************

MG01588.1 	FYYEILNAPDQACHLAKQAFDDAIAE1LDTLSEESYKDSTLIMQLLRDNL0TLWTSSEAE
NCU02806.1	FYYEILNAPDQACHLAKQAFDDAIAE~LDTLSEESYKDSTLIMQLLRDNL0TLWTSSEAE
FG06847.1 	FYYEILNAPDQACHLAKQAFDDAIAE1LDTLSEESYKDSTLIMQLLRDNL0TLWTSSEAE
AN4501.1  	FYYEILNSPDQACHLAKLAFDDAIAE1LDTLSEESYKDSTLIMQLLRDNL0TLWTSSEAE
          	*******:********* ******** *********************** *********

MG01588.1 	P-TASTGHAEAPAAAAATEEKPAEAAAAAEEAPKPAE
NCU02806.1	A-PAAQG-------EAAPEE--PKADAPAAEEPK-AE
FG06847.1 	TSAAGQAEAPKEDAPAAATAEDAPAAPAAAEEPK-AE
AN4501.1  	P--AAEN--------APAEK--KEEAPAAEGEEKPAE
          	.  *.          *..        ..*    *.**
```
